# Anticonvulsant Activity of Hydroalcoholic Extract and Aqueous Fraction of *Ebenus stellata* in Mice

**Published:** 2012

**Authors:** Ayeh Khodaparast, Mohammad Sayyah, Soroush Sardari

**Affiliations:** 1 *Department. of Physiology and Pharmacology, Pasteur Institute of Iran, Tehran, Iran*; 2 *Department of Biotechnology, Research & Science Campus, Azad University, Tehran, Iran *; 3 *Department of Medical Biotechnology, Biotechnology Research Centre, Pasteur Institute of Iran, Tehran, Iran *

**Keywords:** Anticonvulsants, Ebenus stellata, Fabaceae

## Abstract

**Objective(s):**

Fabaceae is the third largest family of flowering plants. Lack of essential oils in the plants of this family can be considered as an advantage and can favor them in search for safe and antiepileptic medicines. The effects of Fabacea family plants including* Ebenus stellata (E. stellata), Sophora alopecuroides* and *Caesalpinia gilliiesii* were evaluated in pentylenetetrazole (PTZ) and maximal electroshock (MES) seizure tests.

**Materials and Methods:**

The hydroalcoholic extracts were obtained by percolation of 100 g aerial parts of each plant in 900 ml ethanol 80%. Acute toxicity of the extracts was assessed. Non-toxic doses of the extracts were injected to the mice intraperitoneally (i.p.) and occurrence of clonic seizures induced by PTZ (60 mg/kg, i.p.) or tonic seizures induced by MES (50 mA, 50 Hz, 1 sec), were monitored up to 30 min after each administration. The anticonvulsant extract was then fractionated by dichloromethane and water. Phytochemical screening of the effective extract was also carried out by thin layer chromatography to verify active constituents.

**Results:**

Among the extracts used, only *E. stellata* had no toxicity and inhibited clonic seizures in a significant and dose-dependent (3-7 g/kg) manner with ED_50_ of 4 g/kg. Fractionation of the extract resulted in dose-dependent (1-5 g/kg) anticonvulsant activity, which was observed in aqueous fraction with ED_50_ of 1.74 g/kg. Phytochemical screening revealed the presence of terpens/sterols, alkaloids, flavonoids, tannin and saponins in the extract.

**Conclusion:**

The presence of anticonvulsant compounds in *E. stellata *suggests further activity-guided fractionation and analytical studies to find the potential of this plant as a source of anticonvulsant agents.

## Introduction

Epilepsy is the third most common neurological disorder after stroke and Alzheimer's disease (1). Current available anticonvulsant drugs are able to efficiently control epileptic seizures in about 50% of the patients; 25% of the cases may show improvement, whereas the rest of the patients do not benefit significantly (2). Furthermore, undesirable side effects of the drugs used clinically often render treatment difficult; so that a demand for new types of anticonvulsants exists. One of the approaches to search for new antiepileptic drugs is the investigation of naturally-occurring compounds, which may belong to new structural classes.

Fabaceae or Leguminosae is a large and economically important family of flowering plants, which is commonly known as the legume family, pea family or bean family. *Fabaceae *is the third largest family of flowering plants, behind Orchidaceae and Asteraceae (3). Several plants from Fabaceae family including *Tetrapleura tetraptera,* *Albizia lebbeck, Sesbania grandiflora, Butea monosperma, Afrormosia laxiflora, Erythrina velutina, Erythrina mulungu, Sutherlandia frutescens, Vicia faba, Astragalus mongholicus*, and *Glycyrrhiza glabra* have shown anticonvulsant activity in animal models (4-15). 

Essential oils often have high toxicity and narrow therapeutic indices. Furthermore, their particular chemical structure has low potential for modification, which renders them unsuitable candidates for drug design. Most of the plants of Fabaceae family have no or negligible amount of the essential oils. This can be considered as an advantage and can favor the plants of this family in search for safe and effective medicines pertaining to new structural classes. 

In this study the possible anticonvulsant and toxic effects of three plants of Fabaceae family including *Ebenus stellata, Sophora alopecuroides* and *Caesalpinia gilliiesii*, were evaluated in mice. 

## Materials and Methods

***Plant materials***

The plants were collected in May 2008*. E. stellata *was collected from Maharloo lake in southeast of . *C. gilliiesii* was collected from . *S. alopecuroides* was collected from sides of Natanz on Isfahan road. The plants were authenticated and the voucher specimens (No.74-8, 74-87 and 85-6, respectively) were deposited in the Herbarium of Pasteur Institute of Iran, Tehran.

***Chemicals***

 Pentylenetetrazole (PTZ), phenytoin sodium and ethosuximide were purchased from Sigma-Aldrich (). Tween 80, dimethyl sulfoxide (DMSO), ethanol, dichloromethane, antimony trichloride, dragendorrf's reagent, potassium hydroxide, glacial acetic acid, vanillin, sulphuric acid, ferric chloride, hydrochloric acid and sodium hydroxide were purchased from Merck (Germany). PTZ, phenytoin sodium and ethosuximide were dissolved in saline solution (0.9% w/v). The extract and its fractions were dissolved in Tween 80 (25%): DMSO (2:1v/v) mixture and used freshly. 

***Extract preparation***

One hundred grams of air-dried aerial parts of each plant were grounded and extracted at the room temperature for 48 hr by percolation method using 80% ethanol (900 ml). The extracts were then concentrated with a rotary evaporator apparatus (IKA-RV 05 basic, Germany) at temperature not exceeding 50 °C. The yields of the extracts were 45% (w/w). The extracts were stored at 4 °C throughout experiments. 

***Fractionation***

**Figure 1:**
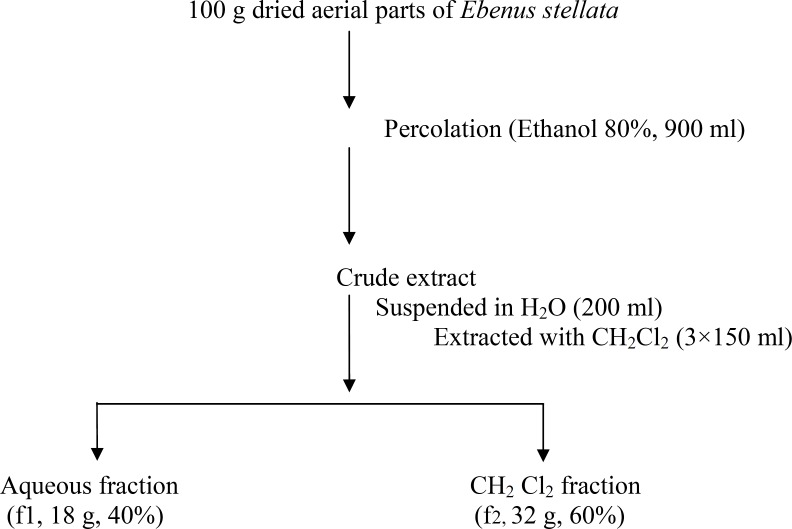
Flow diagram of fractionation of the crude extract obtained from the aerial parts of* Ebenus stellata*

The crude extract of *E. stellata *was suspended in 200 ml distilled water and extracted with dichloromethane (three times, 150 ml each). The dichloromethane and the aqueous fractions were collected separately, dried by rotary evaporator at 40 °C and stored at 4 °C throughout experiments (Figure 1). 

***Preliminary phytochemical screening***

The crude extract of *E. stellata *was screened for the presence of triterpens/sterols, alkaloids, flavonoids, anthraquinones, anthrones, coumarines, valepotriates, essential oil and tannins by thin layer chromatography using silica gel G (Merck) plates of 0.25 mm thickness (16). The extract was dissolved in Tween 80 (25%): DMSO (2:1v/v). Development was carried out with ethyl acetate: methanol: water (100: 13.5: 10 v/v/v) and ethyl acetate: toluene (93: 7). After development, the plates were sprayed with the following reagents for detection of the respective classes of compounds: antimony trichloride (triterpens/sterols), Dragendorrf's reagent (alkaloids), potassium hydroxide (anthraquinones, anthrones and coumarins), hydrochloric acid and glacial acetic acid mixture (valpotriates), vanillin and sulfuric acid mixture (essential oil), and ferric chloride (tannins). Reagents were prepared according to Stahl method (17). Detection was carried out visually in visible light and under UV light (λ = 365 nm).

***Animals***

A total number of 350 male NMRI mice (20-28 g, Pasteur Institute of Iran) were used. The animals ere housed in standard cages (ten mice in each cage) with free access to food (standard laboratory rodent's chow) and water. The animal room temperature was maintained at 23±1 °C with a 12 hr light/12 hr dark cycle (light on from 06:00 a.m.). The study was approved by the Ethics Committee of Pasteur Institute of Iran and conforms to the European Communities Council Directive of 24 November 1986 (86/609/EEC). All animal experiments were carried out in such a way to minimize the number of animals and their suffering. Each animal was tested once. All injections were done intraperitoneally (i.p.) in volume of 0.1 ml/10 g of mice body weight.

***Acute toxicity***

Mice (thirteen different groups, ten mice in each group) were treated i.p. with the solvent of the extracts (10 ml/kg), different doses of the extracts (*C. gilliiesii*: 0.5, 1 and 4 g/kg, *S. alopecuroides*: 0.2, 0.5 and 1 g/kg; *E. stellata*: 5, 6 and 7 g/kg) or the fractions (f1: 5 and 7 g/kg, f2: 3 g/kg). The mortality rate was recorded after 24 hr.

**Figure 2:**
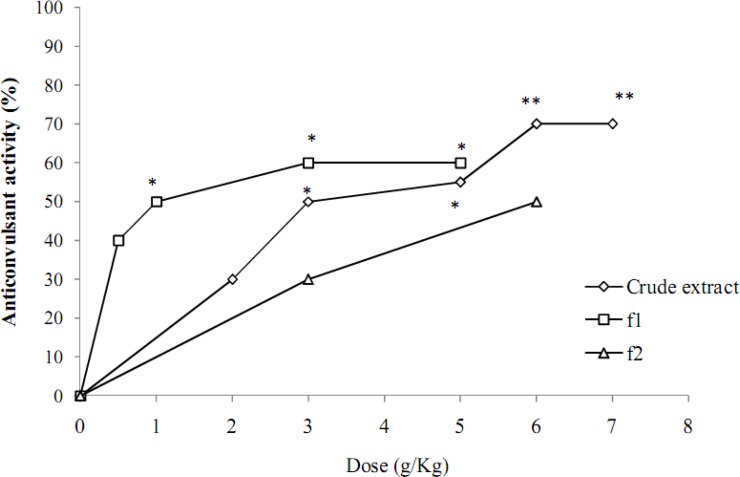
Effect of hydroalcoholic extract and fractions (f1: aqueous fraction, f2: dichloromethane fraction) of* Ebenus stellata* on clonic seizures induced by pentylenetetrazole in mice.

n= 10. ٭*P*< 0.05 and ٭٭*P*< 0.01 compared to control value

***PTZ-induced seizures***

 The minimal i.p. dose of PTZ at which 99% of the animals showed general clonus was determined by a dose-percent effect curve (18). General clonus was considered as the criteria of clonic seizure, which was characterized by clonus of four limbs with transient loss of righting reflex (19). The extracts (*C. gilliiesii*: 0.5 g/kg, *S. alopecuroides*: 0.2 g/kg; *E. stellata*: 2, 3, 5, 6 and 7 g/kg) and fractions (f1: 0.5, 1, 3 and 5 g/kg, f2: 3 and 6 g/kg), the solvent of the extracts and the fractions Tween 80 (25%): DMSO (2:1, v/v) 10 ml/kg, as control), ethosuximide (150 mg/kg, as positive control) and saline (10 ml/kg, as control) were injected to the mice (sixteen different groups, ten animals in each group). After 30 min, PTZ (60 mg/kg) was injected to the animals. If no general clonus occurred during a 30-min period of observation, the animals were considered protected. 

***MES-induced seizure***

 Electro-convulsive shock, inducing hind limb tonic extension (HLTE) in 99% of the animals (18) was determined by a current intensity-percent effect curve. The electrical stimulus (50 mA; 50 Hz; 1-sec duration) was applied (15) through ear-clip electrodes (using a stimulator apparatus (MGH-777, Development of Electronic Industry, Iran). Six groups of 10 mice, each were pretreated i.p. with the different doses of the extracts (*C. gilliiesii*: 0.5 g/kg, *S. alopecuroides*: 0.2 g/kg; *E. stellata*: 7 g/kg), phenytoin (25 mg/kg, as positive control), saline (10 ml/kg, as control) and the solvent of the extracts (10 ml/kg, as control). After 30 min the animals received transauricular electroshock. If no HLTE was observed within 10 sec after delivery of the electroshock, the animals were considered protected. 

***Data analysis***

The dose of the extract required to produce an anticonvulsant effect (ED_50_) in 50% of the animals was calculated by the method of Litchfield and Wilcoxon (18) using a commercial computer program (GRAPHPAD INSTAT 3, version 2003). Data obtained from convulsive tests were expressed as the percentage of the animals showing convulsions and Fisher's exact test was used to analyze the data. *P-*value less than 0.05 was the critical criterion for statistical significance.

## Results

***Mortality***

The crude extracts of *S. alopecuroides* and* C. gilliiesii* showed lethal effects at the doses of 0.5 and 1 g/kg, respectively. However, *E. stellata* crude extract and fractions had no toxicity up to the dose of 7 g/kg (Table 1).

**Figure 3:**
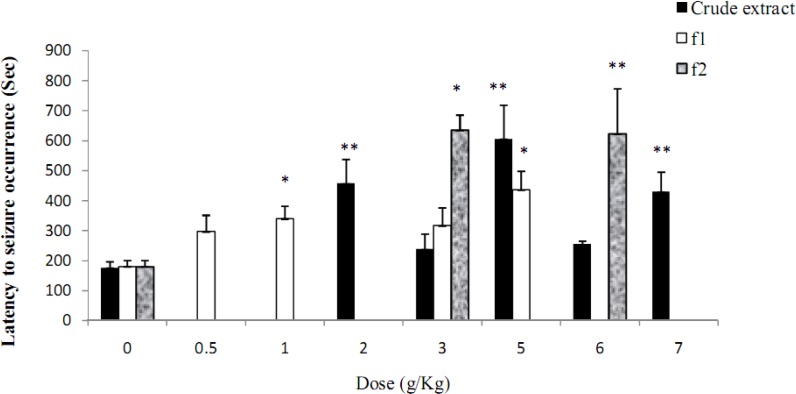
Effect of hydroalcoholic extract and fractions (f1: aqueous fraction, f2: dichloromethane fraction) of* Ebenus stellata* on latency to occurrence of clonic seizures induced by pentylenetetrazole in mice. n=10. ٭*P*<0.05 and ٭٭*P*<0.01 compared to control value.

***Anticonvulsant activity***

The hydroalcoholic extracts of *S. alopecuroides* and *C. gilliiesii* up to the maximum non-toxic doses did not show any anticonvulsant effect against seizures induced by PTZ and MES (Tables 2 and 3). The extract of *E. stellata *up to the dose of 7 g/kg did not show any anticonvulsant effect against tonic seizures induced by MES (Table 3). It however inhibited clonic seizures induced by PTZ and ED_50_ of 4 g/kg was obtained for the extract (Figure 2, Figure 3). 

Fractionation of *E. stellata *crude extract by dichloromethane and water led to increase in anticonvulsant potency that was observed in aqueous phase and ED_50_ of 1.74 g/kg was obtained (Figure 2, Figure 3).

**Table 1:** Acute toxicity of *Ebenus stellata, Sophora alopecuroides *and *Caesalpinia gilliiesii* hydroalcoholic extracts and fractions in mice

Treatment	Dose	Incidence of mortality %
Control	10 ml/kg	0
*C. gilliiesii*	0.5 g/kg	0
*C. gilliiesii*	1 g/kg	40
*C. gilliiesii*	4 g/kg	90***
*S. alopecuroides*	0.2 g/kg	0
*S. alopecuroides*	0.5 g/kg	50*
*S. alopecuroides*	1 g/kg	100***
*E. stellata*	5 g/kg	0
*E. stellata*	6 g/kg	0
*E. stellata*	7 g/kg	0
f1	5 g/kg	0
f1	7 g/kg	0
f2	3 g/kg	0

n=10. ٭*P*< 0.05 and ٭٭٭*P*< 0.001 compared to control value. Control: Tween 80 (25%): DMSO (2:1, v/v), solvent of the extract and the fractions. f1: aqueous fraction of *Ebenus stellata* hydroalcoholic extract, f2: dichloromethane fraction of *Ebenus stellata* hydroalcoholic extract.

**Table 2:** Effect of hydroalcoholic extracts of* Sophora alopecuroides* and *Caesalpinia gilliiesii* on clonic seizures induced by pentylenetetrazole in mice.

Treatment	Dose	Incidence of clonic seizures (%)	Latency to occurrence of clonic seizures (sec)
Control 1	10 ml/kg	100	176.2 ± 40.3
Control 2	10 ml/kg	90	180.2 ± 20.3
Ethosuximide	25 mg/kg	0***	-
*C. gilliiesii*	0.5 g/kg	100	344.7 ± 54.8*
*S. alopecuroides*	0.2 g/kg	100	176.8 ± 27.3

n=10, ٭*P*< 0.05 and ٭٭٭*P*< 0.001 compared to control value. Control 1: Saline, solvent of Ethosuximide; control 2: Tween 80(25%): DMSO (2:1, v/v), solvent of the extracts and fractions.

***Preliminary phytochemical analysis***

The constituents of the crude extract of *E. stellata *are demonstrated in Table 4. The extract contains triterpens/sterols, flavonoids, alkaloids, saponins, and tannin. Coumarins, anthrones, valepotriates, anthraquinones and essential oil were absent in the extract.

## Discussion

PTZ and MES are the most commonly used preliminary tests for screening of potential anticonvulsant drugs. The MES test is considered to be a predictor of likely therapeutic efficacy against generalized tonic-clonic seizures whereas the PTZ test represents a valid model for human generalized myoclonic and also absence seizures (19). 

Fabaceae, which is the third largest family of flowering plants, has been widely studied in search for safe and effective antiepileptic medicines (4-15). The genus* Ebenus* belongs to this family and has more than 100 species (3). However, there is no report regarding the biological effects of the plants in this genus, such as *E. stellata*. The present study is the first report in this regard demonstrating that the crude hydroalcoholic extract of *E. stellata* possesses protective effect against clonic seizures induced by PTZ. Fractionation of the extract by dichloromethane resulted in 2.5 times augmentation in the anti-seizure potency, as ED_50_ of 1.74 g/kg was obtained for the aqueous fraction. This finding indicates that fractionation has been capable of separating the anticonvulsant components from the crude extract. It seems that acceptable potency as well as no toxicity of the aqueous fraction makes it worthy for further studies. 

Our results indicated that the active anticonvulsant principle(s) present in *E. stellata* are polar compounds, since the activity was observed in aqueous fraction and not in the dichloromethane fraction. The phytochemical tests performed in this study showed the presence of triterpens/sterols, alkaloids, flavonoids, tannin, and saponins in the crude extract of *E. stellata*. The anticonvulsant activity of triterpens (20), flavonoids (21), saponins (22-23) and alkaloids (24) has been demonstrated previously. Therefore, the anticonvulsant activity of the extract and its aqueous fraction could be attributed to the activity of triterpens, flavonoids and alkaloids present in the plant. 

It has been proved that reduction of T-type Ca^2+^ currents by drugs such as ethosuximide can prevent seizures induced by PTZ (25).

**Table 3:** Effect of *Ebenus stellata; Sophora alopecuroides;* *Caesalpinia gilliiesii* hydroalcoholic extracts on tonic seizures induced by maximal electroshock in mice

Treatment	Dose	Incidence of tonic seizures (%)
Control 1	10 ml/kg	100
Control 2	10 ml/kg	90
Phenytoin	150 mg/kg	0***
*C. gilliiesii*	0.5 g/kg	100
*S. alopecuroides*	0.2 g/kg	100
*E. stellata*	7 g/kg	100

n=10, ٭٭٭*P*< 0.001 compared to control value. Control 1: Saline, solvent of phenytoin; control 2: Tween 80 (25%): DMSO (2:1, v/v), solvent of the extracts.

**Table 4:** Components of the hydroalcoholic extract of the leaves of *Ebenus stellata*

Compound	Hydroalcoholic extract
Triterpens/sterols	+
Alkaloids	+++
Flavonoides	++
Saponins	+
Tannin	++
Anthrones	-
Anthraquinones	-
Coumarines	-
Valepotriates	-
Essential oil	-

+: positive; -: negative

Drugs that enhance gamma amino butyric acid-type A (GABA_A_) receptor-mediated inhibitory neurotransmission, such as benzodiazepines and phenobarbital can also prevent PTZ-induced seizures (26). Furthermore, activation of N-methyl-D-aspartate receptor appears to be involved in the initiation and generalization of the PTZ-induced seizures (27). Accordingly, drugs that block glutamatergic excitation mediated by NMDA receptor such as felbamate have anticonvulsant activity against PTZ-induced seizures (26). Flavonoids, as one of the major components present in *E. stellata*, are reported to potentiate GABA-induced currents in native GABA_A_ receptors expressed in cortical neurons (28) and also to selectively modulate GABA_A_ receptor subtypes (29-30). Moreover, flavonoids block NMDA receptors in a concentration-dependent manner (31-32). 

Alkaloids, the other main component found in *E. stellata*, have shown anticonvulsant activity against seizures induced by kainic acid, PTZ and bicuculine (33-34). Terpenoids have also NMDA receptor-blocking (35) and GABA_A_ receptor positive-modulation properties (30). Finally, saponins have been reported to protect NMDA-induced neuronal death via a competitive interaction with the glycine-binding site of NMDA receptors in cultured hippocampal neurons (36). Saponins also block GABA specific transporters selectively, which results in inhibition of GABA uptake (37) and propounds saponin compounds as anticonvulsant agents (38). Collectively, these reports provide some experimental evidence for the involvement of the glutamatergic and GABAergic system in the anticonvulsant action of *E. stellata*. 

## Conclusion

The hydroalcoholic extract of *E. stellata *and its aqueous fraction possess protective effect against PTZ-induced clonic seizures. Acceptable potency and lack of acute toxicity suggest further activity-guided fractionation and analytical studies to explore the anticonvulsant agents present in this plant.
